# Meal timing and its role in obesity and associated diseases

**DOI:** 10.3389/fendo.2024.1359772

**Published:** 2024-03-22

**Authors:** Beeke Peters, Janna Vahlhaus, Olga Pivovarova-Ramich

**Affiliations:** ^1^ Research Group Molecular Nutritional Medicine and Department of Human Nutrition, German Institute of Human Nutrition Potsdam-Rehbruecke, Nuthetal, Germany; ^2^ German Center for Diabetes Research (DZD), München, Germany; ^3^ University of Lübeck, Lübeck, Germany; ^4^ Department of Endocrinology and Metabolism, Charité – Universitätsmedizin Berlin, Corporate Member of Freie Universität Berlin, and Humboldt-Universität zu Berlin, Berlin, Germany

**Keywords:** meal timing, circadian clock, chrononutrition, precision nutrition, obesity, metabolic diseases

## Abstract

Meal timing emerges as a crucial factor influencing metabolic health that can be explained by the tight interaction between the endogenous circadian clock and metabolic homeostasis. Mistimed food intake, such as delayed or nighttime consumption, leads to desynchronization of the internal circadian clock and is associated with an increased risk for obesity and associated metabolic disturbances such as type 2 diabetes and cardiovascular diseases. Conversely, meal timing aligned with cellular rhythms can optimize the performance of tissues and organs. In this review, we provide an overview of the metabolic effects of meal timing and discuss the underlying mechanisms. Additionally, we explore factors influencing meal timing, including internal determinants such as chronotype and genetics, as well as external influences like social factors, cultural aspects, and work schedules. This review could contribute to defining meal-timing-based recommendations for public health initiatives and developing guidelines for effective lifestyle modifications targeting the prevention and treatment of obesity and associated metabolic diseases. Furthermore, it sheds light on crucial factors that must be considered in the design of future food timing intervention trials.

## Introduction

The circadian clock is an endogenous timing program that structures physiology and behavior according to the time of day, representing an adaptation to the 24-h light–dark cycle and corresponding environmental changes dictated by the Earth’s rotation (1). The circadian clock plays an essential role in metabolic processes. The organism’s ability to assimilate nutrients, mobilize these nutrients, and discard metabolic waste at specific times of the 24-h day is primed by daily rhythms in the function of numerous genes (2, 3). The desynchronization of the circadian clock associated with the modern 24/7 lifestyle increases the risk for several diseases, such as obesity, type 2 diabetes mellitus, and cardiovascular diseases (2, 4). Food intake is one of the most important *zeitgebers* (3, 5), and therefore, meal timing, together with food composition and calorie intake, is a crucial factor affecting metabolic health (4, 6). Nevertheless, eating times widely differ in the population, and time windows for food intake can be large, exceeding 14 h a day (7). Environmental and lifestyle factors, such as eating around the clock or shift work, which is accompanied by delayed or night eating, increase the risk of metabolic diseases (8).

Therefore, one can hypothesize that social structures such as planned break times at work and social obligations (e.g., regular family meal timing or religious fasting) can influence eating times from small families up to entire population groups. Additionally, with regard to different chronotypes in the population, meaning that each individual person has a circadian preference for performance and activity (9), fixed social structures and environmental conditions must be given more attention. In addition, precision nutrition, i.e., the individualization of eating times or the adaptation of meal plans to the individual circadian rhythms (chronotype), could be an approach to reduce or even eliminate desynchronization. Therefore, it is necessary to differentiate between hereditary factors and adaptable environmental factors linked to the internal circadian clock influencing meal timing.

## The interplay between internal clock and metabolism

The function of the circadian clock in mammals has been previously described in several excellent reviews (10–12). This complex, interlinked hierarchical timing system is responsible for approximately 24-h rhythms in mammals, e.g., in humans, regulating corresponding day vs. night behavior, mainly through the central clock in the brain, but also via peripheral clocks in different tissues and organs (6). In this way, the circadian clock rhythmically coordinates biological processes to occur at the correct time to maximize the fitness of an individual. This enables the body to adapt to changes during the day and night, for example, by regulating eating rhythms or expression levels of certain genes, leading to corresponding circadian rhythms in metabolic function (4). Corresponding oscillations of processes or functions are designated as circadian or diurnal rhythms. The term “circadian rhythms” generally refers to ~24-h oscillations that occur in the absence of external timing cues (i.e., are endogenous). Diurnal (daily) rhythms observed during real-life conditions result from interactions between the internal clock and timing cues, which include light and food intake (2).

**Figure 1:**
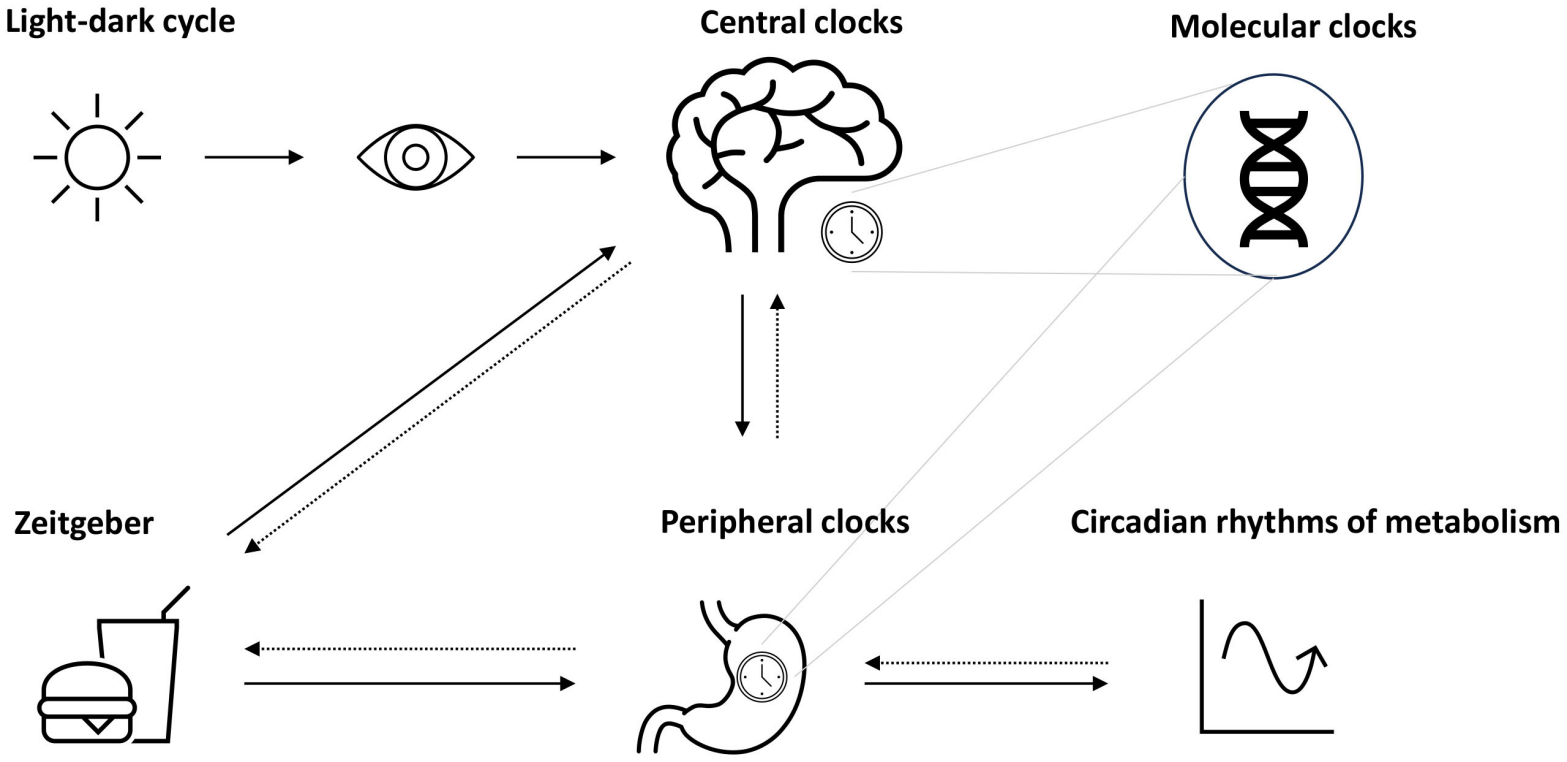
Circadian system and metabolic rhythms. The circadian system is coordinated by the suprachiasmatic nucleus (SCN) as the pacemaker, which is entrained by light (symbolized as sun). Together with further secondary clocks, the SCN influences the expression of gene making up molecular clocks in different tissues (peripheral clocks). Apart from light, further time cues (*zeitgeber*) such as food intake or physical activity can influence the circadian rhythms, e.g., through the modulation of metabolism, which may act as a feedback to adjust central and peripheral clocks.

One crucial oscillator in the clock network is the so-called master clock or pacemaker, also known as the suprachiasmatic nucleus (SCN), located in the hypothalamus and entrained by light (4, 6) (**Figure 1**). The mechanism of the central molecular clock is based on transcriptional and post-translational feedback loops through the activation and repression of the gene’s own transcription (1, 3, 13). The master clock orchestrates the timing of physiological processes according to the 24-h light–dark cycles (14). Identical functioning peripheral clocks are present in almost every tissue (15). Metabolically active tissues and organs, including liver, gut, adipose tissue, and pancreas, are regulated by the circadian system and demonstrate oscillating rhythms underlying synchronization (16). A number of processes involved in digestion, absorption, utilization, and metabolization of food undergo daily rhythms (16). A growing body of evidence leads to the assumption that the maintenance of metabolic homeostasis is based on the interaction of the circadian clocks and metabolism, meaning that the central clock and peripheral clocks are well-coordinated (4).

### Disruption of circadian clock machinery in rodents

Rodent studies have emphasized the crucial role of the internal clock in metabolic regulation on a systemic and organ- or tissue-specific level. Indeed, circadian clock mutant mice or knockout models of individual clock and clock-controlled genes have shown dysfunctions of the metabolic homeostasis (17). For instance, Turek et al. (18) highlighted weakened feeding rhythms, hyperphagia, obesity, hepatic steatosis, adverse changes in fat metabolism, and impaired glycemic control accompanied by hyperglycemia and hypoinsulinemia in *Clock Δ19* mutant mice. Zuber et al. (19) demonstrated an adjustment of renal function up to a diabetes insipidus, accompanied by dysregulated sodium excretion rhythms in *Clock* knockout mice, emphasizing the importance of molecular clock for kidney function. The Per1 transcription factor, encoded by the *Per1* gene in kidney, was identified as crucial for maintaining renal homeostasis (20). Additionally, Clock and Bmal1 transcription factors were recognized for their major role in the recovery regulation from insulin-induced, highlighting their importance in glucose homeostasis (21). Bmal1 showed specific relevance in the peripheral liver clock maintaining fasting glucose homeostasis and glucose clearance (22). Bmal1 and Clock were also found to be essential for pancreatic beta-cell clock function, influencing glucose metabolism, insulin signaling, and insulin secretion (23, 24). Clock protein Cry1 was identified as a regulator for gluconeogenesis in the liver (25). Moreover, the factor Per2 in mice was identified to play an important role in mechanisms related to adipogenesis, inflammation, and insulin sensitivity through direct repression of the nuclear receptor PPARγ (26). *Cry1* and *Cry2* gene deficiency in mice was accompanied by hypertension, leading to the hypothesis that both clock core components are involved in the regulation of blood pressure (27). Rev-erbα was identified as a regulator of lipid metabolism, repressing the transcription of gene coding Apolipoprotein C-III (28). Knockout of the clock gene *Rorα* resulted in severe atherosclerosis and reduced HDL levels in mice fed with an atherogenic diet (29).

## Food intake and metabolic rhythms

To ensure synchronized circadian rhythms, corresponding time cues from the surrounding environment are needed, known as *zeitgeber*, such as light, physical activity, and food intake. Food intake, in particular, is a unique external clock synchronizer as it specifically affects the peripheral clocks of tissues and organs related to food intake, such as the liver, gut, and adipose tissue (15). Meal timing, therefore, entrains or, in other words, synchronizes peripheral circadian rhythms in metabolic tissues independently of the central clock (30). More precisely, in humans and animals, food intake has been proven to entrain the peripheral clocks of liver, heart, and pancreatic tissue (31–33). Under normal conditions, feeding rhythms align with the external light–dark cycle, contributing to the entrainment of peripheral oscillators with the central clock (34). However, food intake during the biological night can uncouple peripheral clocks from the SCN (31, 32), confirming that meal timing acts as a *zeitgeber* for circadian clocks.

### Regulation of food intake and its feedback on metabolism

Historically, it has been thought that food intake is mainly regulated by physiological feedback mechanisms. This internal signaling system is primarily located in the hypothalamus, brain stem, and limbic system (35), where corresponding oropharyngeal, gastrointestinal, blood chemical, tissue metabolic, and other signals are integrated and translated into neuropeptide release (36). The idea is that this system stimulates and inhibits eating to maintain internal homeostasis. Signals can be categorized into short-term regulation, such as gastrointestinal filling, and long-term regulation, for example, feedback signals from adipose tissue (37). Indeed, the complexity of the system underlying food intake provides several new targets/strategies to control appetite, especially at a time when cardiovascular diseases, diabetes, and metabolic syndrome are showing increasing prevalence (38).

### Food intake and circadian rhythms

Body weight control depends on various factors, such as the quantity and quality of food, as well as on meal timing (39). Understanding environmental factors that affect food intake is of great importance, as it seems to play a crucial role in the development of obesity (40). When a time cue, e.g., food intake, occurs at unusual times, this can lead to a conflict between the central and peripheral clocks, resulting in internal desynchronization (41). This occurs through metabolic feedback, which, in the case of food intake, can be the secretion of hormones involved in the regulation of metabolic processes, satiation, and appetite. For example, insulin, glucagon, adiponectin, corticosterone, leptin, chemerin, lipocalin, and visfatin demonstrate diurnal variations typically adapted to food intake throughout the active phase (which is at daytime for humans). Many of these hormones show marked postprandial changes, mirroring the pattern of the food intake (42, 43).

Another feedback mechanism involves microbiota oscillations, which coordinate the activity of diverse metabolic pathways before or after eating events in a 24-h rhythm when meal timing occurs regularly (44). A potential hypothesis for the circadian coordination of these mechanisms is the facilitation of overnight fasts (45). To be precise, hunger and corresponding appetite peaks in the biological evening and morning can be seen as a support for energy balance and, additionally, reduce the risk for awaking during sleep due to hunger (46). Consequently, when these complex interactions are disrupted through mistimed eating, traveling to another time zone, or shift work, so-called chronodisruption, desynchronization between the central and peripheral clocks can occur, leading to impaired metabolic homeostasis and adverse metabolic health effects.

## Mistimed food intake and metabolic disorders

In recent years, several animal and human studies intensively investigated the effects of meal timing on metabolism, specifically examining the effects of mistimed food intake. Long-term desynchronization of circadian rhythms is associated with adverse metabolic health effects, including weight gain, increased body fat, obesity, impaired glucose tolerance, insulin resistance, higher blood pressure, and elevated cholesterol levels, as well as elevated risk for cardiovascular diseases, diabetes, and metabolic syndrome (18, 47–54). These health effects, with regards to meal timing, will be discussed in more detail below, starting with mouse studies followed by human studies.

In mouse studies, feeding animals only during the rest (light) phase resulted in greater weight gain compared to feeding them during the active (dark) phase (55). A replication and extrapolation of this study showed that mice gained even more weight under constant light conditions, but not when restricting the food intake to their “biological night” (56). Additionally, time-restricted feeding was found to compensate for the adverse effects of an unhealthy (high-fat) diet (57). Mistimed food intake in further animal studies has led to increased peripheral inflammation (58).

Similar findings are observed in humans, particularly in shift workers. A systematic review and meta-analysis indicated that despite no differences in total energy intake on dayshift vs. nightshift days, typical adverse effects of mistimed food intake, such as increased risk for obesity, were detected (52). This resulted in the hypothesis that other factors such as meal timing could explain the higher obesity and cardiometabolic disease prevalence among nightshift workers (59). Observational and experimental studies in humans confirm an association between meal timing, weight gain, glucose (in)tolerance, and diabetes (60). For example, later eating times are associated with the development of overweight (61). There are different biological explanations for these relationships. Their understanding could potentially open a new dimension in nutrition science, as most weight loss interventions currently focus on total energy intake rather than meal timing (62).

Further human studies have also focused on breakfast timing and meal regularity (63). Investigations in a female Swedish cohort revealed more food events and a shift towards a later meal timing in women with obesity (64). Along with this, another study stated a longer eating time during the day and quicker eating in women with obesity compared to women without obesity (65). A possible explanation for these associations is that night eating, defined as the intake of the majority of calories after 20:00, is linked to a higher body mass index (BMI), independently of sleep behavior (66–68). A study with 110 participants revealed an association of BMI and adiposity with food intake later in the “biological night”, but not the clock hours, emphasizing that meal timing should be considered relative to the inner clock (69). The latter could explain why some studies have not found associations between meal timing and metabolic outcomes, as the definition “night” is not always aligned with the biological night of an individual, which can vary depending on the chronotype (70).

These findings are supported by further clinical studies, suggesting that late main meal timing hinders intended weight loss (62) or even the therapeutic effect of bariatric surgery. Additionally, several randomized, crossover studies show that eating lunch at a later time reduces resting metabolic rate and equivalent energy expenditure (71, 72). Similar results, indicating an increased risk for weight gain, were observed when the main energy component was consumed in the evening instead of midday (73), showing a connection to impaired insulin sensitivity measured in insulin secretion and glucose tolerance (74). This potentially increased risk for developing a type 2 diabetes (T2D) was also reported when the same meal was consumed in the evening rather than the morning, leading to an increased glycemic/insulinemic response (72). Surprisingly, there was even a resemblance to the postprandial response of a (pre)diabetic patient, despite the subject being healthy (75). Furthermore, later dinner times showed a worsening of postprandial glucose profiles after breakfast on the next day (76). Overall, it is suggested that a low-energy first meal and a high-energy last meal are linked to difficulties in losing weight and impaired glucose metabolism (77). In contrast, the intake of the majority of calories in the morning, such as for breakfast, along with a minority of calories in the evening, has shown metabolically beneficial effects in terms of improving glucose tolerance, which is particularly relevant for T2D patients (78, 79).

Recently, Dashti et al. (80) summarized the adverse cardiometabolic effects of mistimed food intake including (1) development of obesity (42), (2) reduced diet-induced thermogenesis in the evening (81), (3) diminished glucose tolerance due to circadian misalignment resulting from lower insulin sensitivity (82, 83), (4) adverse effects of elevated melatonin on glucose metabolism (84), and (5) an increase in body fat (69). Furthermore, mistimed meal intake resulted in a phase shift in the peripheral clocks of the adipose tissue (30), changes in blood pressure (85), and an increase in inflammatory markers (86).

Over the last 40 years in the United States (US) (1971–2010), a shift in meal timing has been observed through the National Health and Nutrition Examination Surveys (NHANES) (87). In fact, a general later intake for all meals, except dinner and snacks after dinner, was detected (87). This development can be seen as negative, due to the adverse metabolic health effects discussed previously and in the scope of the current obesity pandemic. Therefore, understanding the underlying mechanisms is crucial to adjusting health in terms of diet and making recommendations for disease prevention.

To comprehend these effects, homeostatic and circadian mechanisms regulating food intake must be examined in more detail, e.g., how exactly the circadian rhythms influence hunger and energy expenditure (88). At night, energy expenditure is at its lowest (resting energy expenditure) in contrast to the morning and/or afternoons as shown using a forced desynchrony protocol (89). Hunger, appetite, and food preferences were investigated in another forced desynchrony study using special ratings, revealing a circadian rhythm for hunger with a peak in appetite in the biological morning around 08:00 and evening at 20:00 (46). These hunger rhythms appear to be robust, as they did not show any changes even when meal timing was manipulated (30) or when participants were sleep-deprived (45).

Finally, it is also important to consider the chronotype, which can be understood as a behavior resulting from the circadian system, influenced by genetics, the light–dark cycle, and other environmental factors such as social and personal surroundings. Chronotypes can range from morning to evening preferences, which are divided into early, intermediate, and late chronotypes (90). Extreme chronotypes, especially the late chronotype, might conflict with daily life structure, for example, due to work or school schedule. This applies not only to sleep behavior but also to meal timing preferences, which can vary depending on the individual chronotype. Furthermore, a late chronotype can lead to discrepancies in workday compared to non-workday behaviors, considered a so-called nutritional or meal jet lag, which may result in metabolically adverse events (90).

All in all, these findings suggest obesity as a chronobiological disease. However, the development of overweight is multifactorial, and regular food intake during the active phase needs to be considered in addition to known factors such as energy intake and/or dietary composition (60). Although we consider food intake as a “chronodisrupter” in the case of desynchronization, a change of paradigm towards a more positive term, such as “timekeeper”, offers the therapeutic chance of meal timing interventions, when considering that meal timing synchronized with the cellular activities can lead to an optimized performance of tissues and organs (91).

## Possible mechanisms of meal timing effects

Despite this clear connection between meal timing and cardiometabolic health, the underlying mechanisms remain largely unknown. An important question is whether the effects are solely due to mistimed food intake or possibly due to other changes, such as energy intake, macronutrient composition of the meal, fasting duration, meal frequency, or changes in sleep behavior, accompanying mistimed meals.

It has been believed that individuals who do not eat early in the day tend to consume more energy in the evening; for example, people who skip breakfast tend to consume more calories and meals over the day (41, 92, 93). Therefore, explaining greater fat storage and the corresponding weight gain might be explained by changes in meal frequency. However, this aspect has not been carefully controlled in many studies.

These effects can also be explained through the disruption of the circadian system as it is highly interconnected with food intake (see also the *Regulation of food intake and its feedback on metabolism* section). In particular, circadian variations are mirrored in the energy expenditure and metabolic pattern of healthy individuals when comparing morning to evening. For example, food-induced thermogenesis is generally higher in the morning than in the evening and night (81). Additionally, when consuming the same meal in the evening instead of morning, lower resting metabolic rate as well as an increased glycemic and insulinemic responses occur (72). Beyond that, it has been shown that signaling pathways involving cAMP-response element binding protein (CREB), mechanistic target of rapamycin (mTOR), and adenosine monophosphate-activated protein kinase (AMPK) also play a role in energy expenditure. When restricting food intake to an 8-h period, CREB, mTOR, and AMPK pathway function and their circadian rhythms increase, leading to a higher expression pattern of certain liver genes and improved nutrient utilization (57).

Further mechanisms are the secretion of (an)orexigenic hormones, e.g., leptin, which is released from the adipose tissue proportional to lipid storage (35). Leptin decreases in terms of concentration as a consequence of mistimed eating. Mistimed eating leads to a decrease in leptin concentration, associated with increased food intake and lower energy expenditure, potentially explaining subsequent weight gain. Not only hormonal, but further digestive components required before food intake are controlled by the circadian clocks, which are diminished at night. For example, saliva flow in the mouth or in the gut, stomach acid, and intestinal motility flow decrease at night (94–96). Many of the digestive components, including peptide hormones of the gastrointestinal tract, not only underlie circadian rhythmicity, but also can affect the peripheral clocks and therefore metabolic rhythms, again providing feedback circuits (97).

Circadian clocks in the muscle and fat mass also play an important role in metabolism. For example, muscle protein synthesis rates are highest during the night, making protein intake, recommended for exercise recovery, most effective before sleeping (98). Regarding adipose tissue, mistimed food intake can increase the expression of the inflammatory markers tumor necrosis factor alpha (TNFα) and macrophage-1 antigen (MAC1) in white adipose tissue, similar to the upregulation seen with a high-fat diet (58). Mice lacking cryptochrome (Cry), a photoreceptor that regulates the entrainment by light of the circadian clock, are more susceptible to high-fat diet-induced obesity (99). Additionally, the expression of lipogenic genes is upregulated in the white adipose tissue of these mice, possibly explaining increased insulin secretion and lipid storage (48). Moreover, adipose tissue specific knockout (BMAL1) showed weight gain due to changes in energy regulation and a shift in food intake, emphasizing the interaction of meal timing and adipocyte clocks (100).

Finally, changes due to mistimed food intake have been observed in the diurnal salivary and gastrointestinal microbiota rhythms in terms of diversity and abundance, all underlying the circadian control (101). For example, eating a late main meal inverts the daily rhythm of salivary microbiota diversity, which may have an effect on the host metabolism (44). Furthermore, microbiota regulates body composition via the nuclear-factor, interleukin 3 regulated (NFIL3), which underlies the circadian control and affects the regulation of lipid absorption and export (102). Interestingly, microbial metabolites can affect the central and peripheral clocks and are linked to various processes in anabolism and catabolism of different macronutrients (103). Desynchronization of circadian rhythms due to mistimed food intake has been associated with gut dysbiosis and consequent metabolic and systemic disorders. In obese and elderly women, similar abnormalities in rhythmicity were observed (104).

## Time-of-day-based nutritional strategy: time-restricted eating

An analysis of eating windows from an Indian cohort of 93 healthy individuals, who documented their food intake using phone cameras for 21 days, revealed that half of the cohort had daily eating windows longer than 15 h (105). Indeed, a decile of the cohort even showed eating windows around 20 h due to flexible working times or a partner engaged in shift work (105). The study demonstrated a caloric consumption of more than 30% of total calories in the evening and night, with less than 30% of total calories consumed in the morning (105). Previously, the identification of long eating periods in the population of American adults has shown benefits for people with overweight through a reduction in the length of the eating window, which manifested itself in weight loss, sleep improvement, and increased energy (7).

This concept of shortening meal times has been established for some time and is widely practiced in today’s society for weight management and modulation of eating patterns. In this regard, the approach of time-restricted eating (TRE) has been proven to shorten the eating window. TRE is characterized by intervals of 8 to 10 h during which food is consumed, with variations regarding time of day (106). In this respect, early TRE (eTRE), mid-day TRE (mTRE), and late TRE (lTRE) have been shown to be variations of TRE (106). The term “TRE” refers to a specific eating pattern or eating habit that relates to the timing of food intake (meal timing) and can be applied regardless of calorie restriction (106). Recent human trials on TRE have highlighted numerous beneficial metabolic effects of TRE, e.g., improved mean daily glucose levels (107, 108), fasting glucose levels (108–110), insulin resistance (108, 110, 111) or insulin sensitivity (112, 113), triglyceride levels (107, 109, 114, 115), total cholesterol (115, 116), and LDL cholesterol levels (116). However, results in TRE trials are highly variable, probably because of heterogeneous study designs, eating windows, study cohorts, and differences in calorie intake (117, 118). With regard to possible metabolic TRE effects that goes beyond a simple calorie restriction diets, recent studies investigated whether the combination of calorie restriction and TRE is more effective than a simple calorie restriction. Thus, Jamshed et al. (119) compared a weight loss treatment with energy restriction (ER), but eating longer than 12 h with a time-restricted weight loss treatment (eTRE+ER). The study revealed more positive effects of eTRE+ER regarding weight loss and blood pressure. However, another study by Liu et al. (120) comparing calorie restriction with or without TRE (CR or CR+TRE) in patients with obesity showed no significant differences in weight changes between the groups after 12 months.

Actually, there is little evidence for a recommendation of the TRE eating window, e.g., eTRE, mTRE, or lTRE. However, one randomized crossover trial in men who were at risk for T2D showed improved glycemic responses through TRE independently from clock time, but only demonstrated reductions of fasting glucose in continuous glucose monitoring when TRE was performed from 8 a.m. to 5 p.m (107).. In a recent published systematic review and meta-analysis of 15 TRE studies, the authors concluded that metabolic effects are dependent on the duration of the TRE eating window (121), with anthropometric and cardiometabolic changes being stronger with shorter eating windows (121).

## Factors influencing meal timing

**Figure 2:**
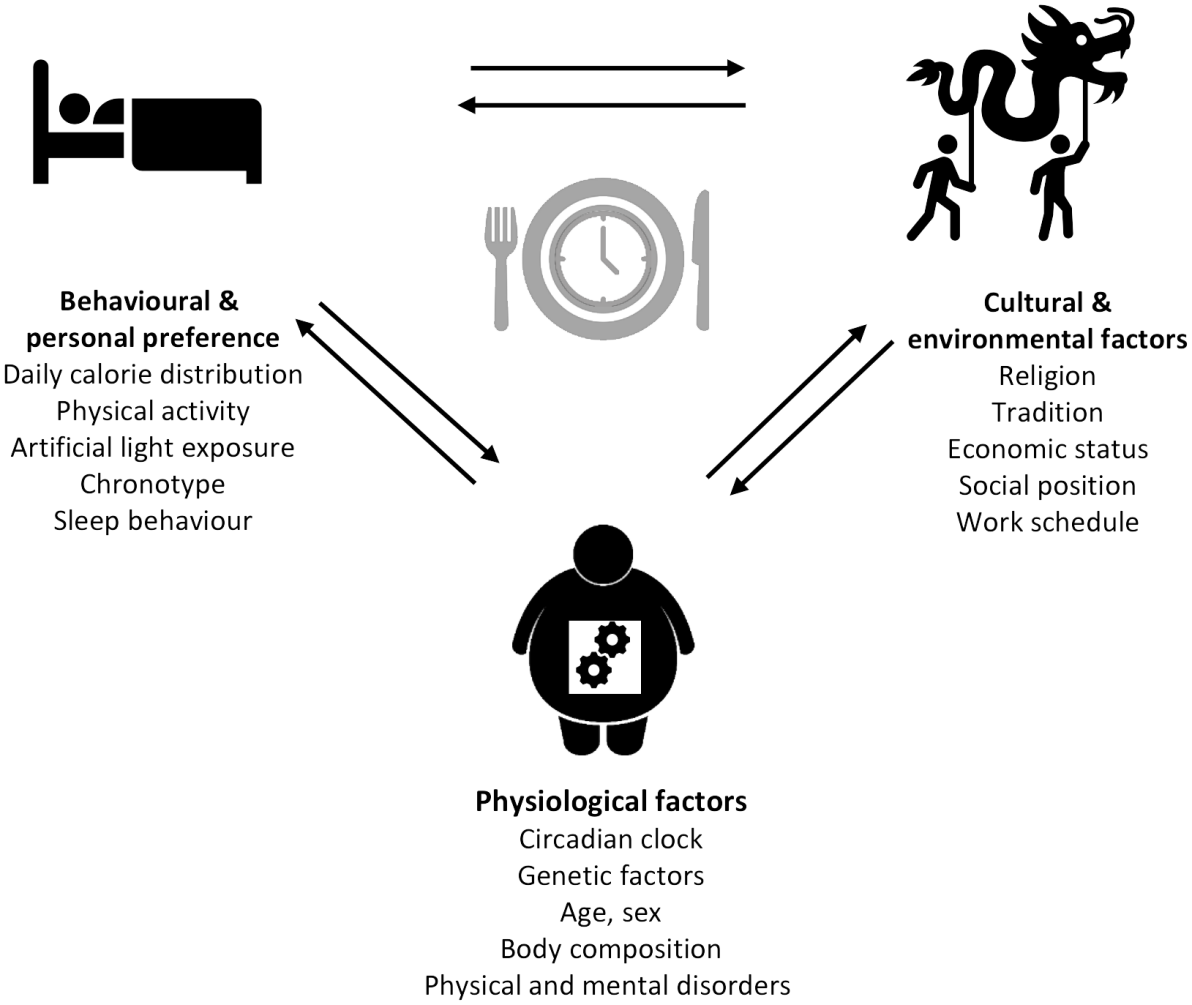
Determinants of meal timing. The factors influencing meal timing range from physiological to behavioral and personal preferences, as well as cultural and environmental factors each comprising different parameters.

The modern lifestyle allows traveling across different time zones, fast food chains enable 24-h food access, and jobs with nighttime working hours open the possibility of working around the clock. Although this does not seem harmful at first and rather indicates possibilities than limitations, a closer look towards the influence of these factors on the circadian system is necessary. Indeed, jetlag through traveling, shift-working, and the constant availability of food, especially with high energy density, and artificial light have been previously described as environmental and lifestyle-associated factors that can lead to chronodisruption (122). A number of social and biological factors influencing meal timing (80) can be divided into different categories: (1) physiological factors (e.g., circadian clock, genetic factors, age, sex, body composition, and physical and mental disorders), (2) behavioral and personal preferences (e.g., daily calorie distribution, physical activity, artificial light exposure, chronotype, and sleep behavior), and (3) cultural and environmental factors (e.g., religion, tradition, economic status, social position, and work schedule) (**Figure 2**). Notably, many of these factors such as age, sex, chronotype, meal composition, physical activity, and others also influence metabolic outcomes, but it is difficult to separate meal-timing-mediated and meal-timing-independent effects on metabolism in free-living individuals. Some of the factors influencing meal timing are discussed in more detail below.

### Sex and age

Next to the circadian system and BMI, sex difference plays a crucial role in metabolic regulation (80). Thus, a meta-analysis in Iranian students including 24 studies revealed a higher prevalence of breakfast-skipping among girls than among boys (123). Sex differences in meal patterns were further described in a study performed in rural areas of Nepal, where the skipping of lunch and daytime-snacking were more frequently in men, than in women (124). Contrary to this, a study performed in 62,298 American adults reported that snacking behavior was more prevalent in women than in men (125). However, the authors also reported a trend in both sexes that a higher intake of snacks unrelated to main meals reduces the number of main meals (125). Moreover, differences in chronotypes (see the *Chronotype* section) dependent on age and sex were described previously in a large American cohort, inducing a higher prevalence of women having earlier chronotypes under the age of 40 years and later chronotypes above 40 years compared to men, possibly due to hormonal changes (126). In addition, there are more investigations pointing out an association of eating pattern in terms of meal timing and age. In fact, skipping breakfast was observed in the 2005–2010 NHANES survey, in which the 20–39 age group was the largest group to report no breakfast in both surveys (92). To conclude, adolescents were found to have the latest chronotypes, even if very early to very late chronotypes can be found in any age group (126).

### Physical and mental disorders

Both physical and mental disorders can affect meal timing, and along with that, some diseases require adaptations of meal timing. One example is the night-eating syndrome where patients tend to suffer from morning anorexia as a result of excessive food consumption (hyperphagia) in the evening, leading to an increase of energy intake mainly in the second half of the day (127). Furthermore, a poorly controlled T2D can lead to a drop in blood glucose levels and may require meals outside of regular meal timing and therefore affecting the eating pattern. Another disease influencing meal timing is liver cirrhosis, where problems in the glycogen storage occur. In this case, patients should change their diet pattern towards a more frequent and therefore smaller food intake to prevent long fasting periods (128). Furthermore, psychiatric or mental disorders may influence dietary intake. Indeed, an association between depression symptoms and nocturnal eating has been described in young adults, indicating an effect on meal timing or *vice versa* (129). Moreover, psychiatric disorders, e.g., binge eating disorder or anorexia nervosa, and addictions, e.g., drug and alcohol addictions, are possible factors influencing meal time patterns.

### Genetic factors

As meal timing can be seen as a potential new risk factor for metabolic diseases, the aim to modify it in order to prevent chronic diseases through weight reduction seems clear (130), but for this, we must gain a better understanding of this complex trait, starting with revealing the genetic and environmental contributions to the individual variability (131, 132). A necessary aim is to understand underlying biological mechanisms and to develop successful public health interventions or effective therapeutic approaches, most probably via personalization (133, 134).

There are limited data regarding the heritability contributing to the food intake and associated parameters such as body weight, which influence overall energy intake (135). However, not only these overarching variables seem to be influenced by genetic factors. There are also emerging data on the heredity of more subtle variables known as the microstructure of food intake, e.g., the frequency, composition, and meal timing, independent of the overall intake (131, 136). Additionally, further twin studies have shown that genetics influence different diet-related phenotypes such as energy and macronutrient intake, dietary patterns, and the intake of specific food items (137). There are even specific physiological variables, such as stomach filling before and after eating (138), or certain metabolic responses (139) that have been proven heritable. Finally, psychological variables such as the subjective rating of hunger and palatability of food have also been found to be influenced by genetic factors (140, 141).

To our knowledge, there have been only two studies that have investigated the heritability of meal timing. One involved 265 identical and fraternal adult twin pairs who recorded 7-day food diaries (142). The other study included 52 Spanish twin pairs who also recorded 7-day food intake and additional sleep diaries (143). Both studies demonstrated highest heritability for breakfast: 24% by De Castro (142) and 56% by Lopez-Minguez et al. (143). This was contrasted by lunch ranging from 18% by De Castro (142) to 38% by Lopez-Minguez et al. (143), whereas the latter team did not detect any heritability for dinner, despite 22% heritability for dinner timing detected by De Castro (142). Furthermore, a high heritability of 64% was shown for the caloric midpoint by Lopez-Minguez et al. (143) with a similar heritability for other phenotypically related behavioral traits such as wake (55%) and bedtimes (38%). These factors even correlated with 85% of co-variance between midpoint and chronotype as well as 90% to wake and 75% to bedtime, implying a common genetic variation. These findings suggest that the heritability of the later meals matches the heritability of evening behavior and is lower than the corresponding heritability for early meals and behavior. This also implies that health interventions should be geared more towards the easier, more modifiable, later meal intake (143).

Genetic variants within core clock genes, which control circadian rhythms of transcription and translation in different cells in the body, are a possible explanation for the heritability of meal timing. In particular, they control the temporality of hunger and appetite, independent of other behaviors (46). Polymorphisms within core clock genes such as PER, CRY, BMAL1, and CLOCK may lead to individual differences in their expression (144). For example, single-gene mutations in the clock gene CRY1 have been shown to confer extreme early or late chronotypes (145).

Furthermore, taking a closer look at the CLOCK gene rs4580704 variant, obese participants carrying the minor G allele had their lunch significantly later than participants carrying major alleles (62). Furthermore, the obesity-related perilipin-1 (PLIN1) gene seems to influence the interaction between meal timing and weight loss: people with rs1052700 amino acid (AA) show less weight loss when eating late compared to TT carriers, where eating late does not influence weight loss at all (146). Genetic influences have been suggested for the night eating syndrome (NES) and sleep-related eating disorders (SRED), which are defined as eating disorders with a preference towards late night eating (147). In addition, the microbiome and its composition, known as the microbiome phenotype, have been shown to account for 97% heritability and could therefore constitute a further important component for the heritability of meal timing (148). Interestingly, the risk allele within melatonin receptor MTNR1B provides a link between late dinner eating and impaired glucose tolerance, whereas in non-risk carriers, this relation was less pronounced (149).

Contrary to these findings, the genome-wide association study (TwinUK) with 2006 participants did not reveal any genetic variant associated with breakfast-skipping possibly because of the modest sample size or incomplete genetic coverage (150). Therefore, it is tempting to elucidate the genes associated with mistimed food intake as these may help to understand the biological mechanisms underlying the regulation of meal timing. Revealing these genes could enable further testing of the relation between meal timing and other related traits, especially towards chronic diseases, in order to identify individuals who are at higher risk of mistimed eating or, on the other side, those who are resistant to timing interventions. Ideally, this could lead to the development of individual diet recommendations in line with genetic preferences.

### Meal composition

Xiao et al. (151) revealed clear associations between carbohydrate and protein intake with obesity, which indicates variations in nutrient response and nutrient requirement around the circadian cycle and therefore reflects health status. Beyond that, the nutrient composition could be a possible factor influencing meal timing. Thus, a study in students who were offered a walnut or an isocaloric gummy candy snack 90 min before eating dinner in the university cafeteria found that they had less food desire compared to the no-snack control group (152). Indeed, the walnut snack with a low glycemic index, low protein, and low polyunsaturated fats showed more positive effects than the gummy candy snack. Moreover, a recent study by Braden et al. (153) showed a lower appetite and higher satiety through micellar casein and pea protein isolate consumption in healthy subjects with a corresponding increase of peptide YY levels when compared with soy protein, but did not show effects on subsequent food intake. With regard to fiber, there is a steady assumption that fiber intake increases satiety. However, a systematic review of 44 publications examined that approximately 61% of the acute fiber treatments did not lead to an enhanced satiety, and beyond that, a relation between fiber type and dose satiety and food intake was not found (154). Since it is not yet clear how nutrients affect meal timing, further future studies on the effects of nutrient timing are needed.

### Chronotype

The chronotype can be described as a complex phenotype representing the individual preference of a person regarding his or her active and sleep time (155). Thus, chronotyping requires the allocation to morning, midday, and evening type (155). However, the chronotype seems to be dependent on environmental factors, which again is affected by mechanisms of innate homeostasis of sleep and circadian rhythms, as shown by a genome-wide association analysis, identifying genomic loci associated to chronotype (156). Owing to a normal distribution of chronotypes, the assumption that common genetic variants in population affect the phenotype arises (157). Indeed, it has been stated that being a *night owl* or a *morning lark* is dependent on the individual genetic makeup (157). Regarding meal timing, it has been shown that the choice of food timing underlies the influence of chronotype (158). Thus, late sleepers, i.e. late chronotypes, consume the more calories later in the day than normal sleepers (67). Furthermore, a recent study that was conducted among Italians during COVID-19 lockdown revealed that evening chronotypes had a higher vulnerability regarding late meal timing, breakfast-skipping, and unhealthy habits in relation to their chrononutrition profiles (158). Along with that, the Sleep Extension Study with 119 participants found that later chronotypes eat breakfast approximately 1.3 h later than early chronotypes and consumed twice as many calories after 20:00 (159). In addition, it has been reported that breakfast-skipping is associated with a poorer glycemic control in patients with T2D and is more present in late chronotypes (160). Furthermore, it has been shown that late chronotypes also have a higher frequency of meals with the largest intake at 19:00 to 21:00 compared to early chronotypes, who have their calories more equally distributed over the day (161).

### Individual lifestyle factors and daily routines

The preferences of people regarding their lifestyle vary widely. Depending on preferences, individuals distribute their daily calorie intake in different ways. Beyond that, personal preferences for a specific hobby can affect eating habits and especially eating times. Thus, the optimal performance and recovery in sports may require an implementation of a special meal timing and composition based on recommendations (98, 162). Along with that, the individuals’ desire for body weight optimization can lead to personal preferences regarding the choice of a specific diet. Indeed, there are several diets, interacting with the natural meal timing as intermittent fasting or TRE, which lay the ground for informed meal timing interventions (80). Moreover, the personal preference for specific foods or beverages can affect meal timing. Thus, it has been shown that energy intake is reduced for obese participants with moderate coffee intake (163), emphasizing caffeine’s effect on appetite and on meal timing. However, another study showed that caffeine intake leads to less energy consumption at breakfast (10.0%), but also demonstrated a shift towards later intake in the day (164). Furthermore, medications and supplements for certain diseases can influence meal timing as they must be taken before a meal, affecting the medication itself on the one hand, but also appetite, which may result in further changes in meal timing (165). Next to the lifestyle factor of caffeine intake, alcohol is a complex dietary component with the potential to affect food intake (166). Thus, the possible short-term appetite-stimulating effects of alcohol can induce a change in meal timing (167). Indeed, the authors of a study involving 282 students concluded that the likeliness towards night eating is higher for those consuming alcohol more frequently (168).

### Artificial light exposure and sleep–wake pattern

In pre-industrial societies, the sleeping routine of people was in harmony with natural light (169). However, in the modern era, light conditions changed with the development of artificial lighting, enabling activities around the clock, e.g., socializing via light-emitting devices, or the possibility of commerce, production, and access to 24-h services, and therefore also leading to shifts in meal timing (170). A possible consequence of this is that light at night and the corresponding longer active compared to rest phase intervals have led to an increase in food intake and therefore excessive weight gain, as shown in rodent studies (56). A significant interaction between food and meal timing was previously described due to an influence of meal timing on sleep timing, and even the sleep timing influence was stronger than the meal timing influence regarding parameters of food intake (171). Apart from wake and sleep timing, long wake periods during the night may lead to a greater possibility for food intake and later eating time (172). Thus, sleep duration influences meal timing and the pattern of food intake (173–175). Social jetlag is a result of sleep depth due to a discrepancy between social and biological times, or rather work and free days (176). Indeed, sleep deprivation of 4 h per night over 5 days leads to an increase in energy consumption of around 533 ± 296 kcal between 22:00 and 03:59 and a reduction of energy intake during 14:59–20:00 compared to baseline (177). Similar findings were reported in another study with sleep restriction, with a 0.8-kg weight gain due to smaller breakfast and a corresponding higher energy intake of 42% after dinner during the nighttime (178). Along with that, another study showed a decrease in the feeling of satiety and fullness in participants who underwent a four-night intervention with 5 h of sleeping (179). This leads to the assumption that sleep deprivation affects meal timing. In contrast, longer sleep episodes correlate with an earlier intake of dinner/the last meal (83) as well as a decreased consumption of free sugars (173).

### Geography, religion, and social status

Geography, ethnicity, and culture seem to affect meal timing. Thus, a survey of 36,020 people in 10 countries across central/northern Europe and the Mediterranean revealed a trend for fewer eating events in Mediterranean countries, with a higher energy intake at lunch (40.0% of daily energy) compared to 20.0% in the central/northern areas (180). In addition, snacking, which has become a prevalent eating style, has been identified as a factor influencing cardiometabolic health (130). In this respect, Mediterranean countries indicate a more mindful handling towards lower snacking behavior due to about approximately 15% snacking compared to 25% to 30% in central/northern European countries (180).

Tradition and religion have been shown to influence meal timing throughout history as well as recently; for example, in the Islamic month of Ramadan, meal timing is restricted to the hours between sunrise and sunset for 1 month (181). Interestingly, overnight fasting has also been shown to vary across Europe, with the shortest period being 09:12 in the Netherlands and 10:54 in the Czech Republic (182). Indeed, the change in meal timing towards eating before and after sunset is associated with changes in circadian rhythms and therefore affects cortisol, melatonin, leptin, or ghrelin levels (183).

Moreover, for centuries, social class has influenced meal timing; for example the former middle class, such as tradesmen and merchants, could eat their meals later because of longer work hours. It has also been shown that people with lower income in the US are more likely to skip breakfast (92) and have fewer eating occasions in general (184).

### Work schedules and social obligations

Work schedules, school times, and opening hours, e.g., from restaurants or supermarkets, are environmental factors that can lead to meal timing adjustment, change in dietary composition, and the amount of eating events. Indeed, it has been shown that weekday and weekend variabilities can influence the number of meals and snacks, which are decreased on weekends in German (68) and US populations (185). The NHANES study (2003–2012), based on data of 11,646 U.S. adults, showed that people consumed more calories and had a poorer diet quality during the weekend, especially on Saturdays, resulting in higher energy, fat, and protein intake; and confirmed previous data from NHANES (1999–2004) that already revealed associations between temporal dietary patters and diet quality (186, 187). However, the differences between weekends and weekdays also occur between (un)employed participants, implying that not only workspace but also cultural and social factors seem to influence food intake (80). Beyond that, the timing factor was identified to be linked to diet quality (187). Furthermore, a cross-sectional analysis of 428 children, assessing differences in eating habits between weekdays and weekends, revealed a more adequate eating habit regarding vegetables, lunch, and snacks on weekdays, therefore indicating the influence of school canteens in promoting health (188). This is another example of the influence of environmental factors on eating behavior. According to this, social meal timing, e.g., the common meal of family members, has recently been discussed as an important factor for metabolic health (189). Moreover, weekday–weekend variability was demonstrated by Gill and Panda (7) who observed a delay of 1 h of breakfast on weekends compared to weekdays, contrary to the mean last caloric intake, most probably dinner, which was more commonly advanced. Moreover, a trend towards later breakfast as well as earlier dinner and less frequent meals with a longer eating window on weekends compared to weekdays has been shown by other smaller studies from the US (159, 161).

In modern society, not everyone has a 9 a.m.-to-5 p.m. job with regular free weekends. Indeed, differences in meal timing can be caused by shift work, 24-h services, or irregular part-time work. Children are already subjected to external influences, e.g., the holiday season. Regarding this, Mohd Azmi et al. (190) discuss several abnormalities of eating behavior in shift workers, e.g., the NES (191), increased food cravings (192), and the difficulty to follow typical eating patterns within rotational shifts (193).

## Conclusion and outlook

In this review, we provided a comprehensive overview of the crucial role of meal timing for metabolic health and the development of obesity and associated diseases. Factors influencing meal timing are very complex and include both internal genetic and external environmental and behavioral factors. They often influence each other directly or indirectly, thus presenting a challenge for the application of meal timing targeting strategies. Nevertheless, environmental factors, e.g., lifestyle, working schedules, and social obligations, are modifiable and can change during a life span. Thus, a precision nutrition approach taking as many internal and external meal time factors as possible into account could be feasible and effective. However, the genetic component needs further investigation in order to understand the metabolic consequences of mistimed food intake.

## Author contributions

JV: Writing – original draft, Visualization. BP: Writing – review & editing, Writing – original draft. OP-R: Writing – review & editing, Writing – original draft, Supervision, Resources, Project administration, Funding acquisition, Conceptualization.
